# Toward a Domain-Overarching Metadata Schema for Making Health Research Studies FAIR (Findable, Accessible, Interoperable, and Reusable): Development of the NFDI4Health Metadata Schema

**DOI:** 10.2196/63906

**Published:** 2025-05-21

**Authors:** Haitham Abaza, Aliaksandra Shutsko, Sophie A I Klopfenstein, Carina N Vorisek, Carsten Oliver Schmidt, Claudia Brünings-Kuppe, Vera Clemens, Johannes Darms, Sabine Hanß, Timm Intemann, Franziska Jannasch, Elisa Kasbohm, Birte Lindstädt, Matthias Löbe, Katharina Nimptsch, Ute Nöthlings, Marisabel Gonzalez Ocanto, Tracy Bonsu Osei, Ines Perrar, Manuela Peters, Tobias Pischon, Ulrich Sax, Matthias B Schulze, Florian Schwarz, Carolina Schwedhelm, Sylvia Thun, Dagmar Waltemath, Atinkut A Zeleke, Wolfgang Müller, Martin Golebiewski

**Affiliations:** 1 Scientific Databases and Visualization Heidelberg Institute for Theoretical Studies (HITS) Heidelberg Germany; 2 Information Centre for Life Sciences German National Library of Medicine (ZB MED) Cologne Germany; 3 Core Facility Digital Medicine and Interoperability Universitätsmedizin Berlin Berlin Institute of Health at Charité Berlin Germany; 4 Institute for Community Medicine University Medicine Greifswald Greifswald Germany; 5 Department of Biometry and Data Management Leibniz Institute for Prevention Research and Epidemiology - BIPS Bremen Germany; 6 Department of Medical Informatics University Medical Center Göttingen Georg-August-University Göttingen Germany; 7 Department of Molecular Epidemiology German Institute of Human Nutrition Potsdam-Rehbruecke Nuthetal Germany; 8 Institute for Medical Informatics, Statistics and Epidemiology University Leipzig Leipzig Germany; 9 Molecular Epidemiology Research Group Max-Delbrück-Center for Molecular Medicine in the Helmholtz Association (MDC) Berlin Germany; 10 Department of Nutrition and Food Science University of Bonn Bonn Germany

**Keywords:** FAIR, findability, epidemiology, data sharing, public health, health research, metadata standards, interoperability development, structure, implementation, epidemiological, data models, accessibility, reusability, tool, clinical trial, nutrition, chronic diseases

## Abstract

**Background:**

Despite wide acceptance in medical research, implementation of the FAIR (findability, accessibility, interoperability, and reusability) principles in certain health domains and interoperability across data sources remain a challenge. While clinical trial registries collect metadata about clinical studies, numerous epidemiological and public health studies remain unregistered or lack detailed information about relevant study documents. Making valuable data from these studies available to the research community could improve our understanding of various diseases and their risk factors. The National Research Data Infrastructure for Personal Health Data (NFDI4Health) seeks to optimize data sharing among the clinical, epidemiological, and public health research communities while preserving privacy and ethical regulations.

**Objective:**

We aimed to develop a tailored metadata schema (MDS) to support the standardized publication of health studies’ metadata in NFDI4Health services and beyond. This study describes the development, structure, and implementation of this MDS designed to improve the FAIRness of metadata from clinical, epidemiological, and public health research while maintaining compatibility with metadata models of other resources to ease interoperability.

**Methods:**

Based on the models of DataCite, ClinicalTrials.gov, and other data models and international standards, the first MDS version was developed by the NFDI4Health Task Force COVID-19. It was later extended in a modular fashion, combining generic and NFDI4Health use case–specific metadata items relevant to domains of nutritional epidemiology, chronic diseases, and record linkage. Mappings to schemas of clinical trial registries and international and local initiatives were performed to enable interfacing with external resources. The MDS is represented in Microsoft Excel spreadsheets. A transformation into an improved and interactive machine-readable format was completed using the ART-DECOR (Advanced Requirement Tooling-Data Elements, Codes, OIDs, and Rules) tool to facilitate editing, maintenance, and versioning.

**Results:**

The MDS is implemented in NFDI4Health services (eg, the German Central Health Study Hub and the Local Data Hub) to structure and exchange study-related metadata. Its current version (3.3) comprises 220 metadata items in 5 modules. The core and design modules cover generic metadata, including bibliographic information, study design details, and data access information. Domain-specific metadata are included in use case–specific modules, currently comprising nutritional epidemiology, chronic diseases, and record linkage. All modules incorporate mandatory, optional, and conditional items. Mappings to the schemas of clinical trial registries and other resources enable integrating their study metadata in the NFDI4Health services. The current MDS version is available in both Excel and ART-DECOR formats.

**Conclusions:**

With its implementation in the German Central Health Study Hub and the Local Data Hub, the MDS improves the FAIRness of data from clinical, epidemiological, and public health research. Due to its generic nature and interoperability through mappings to other schemas, it is transferable to services from adjacent domains, making it useful for a broader user community.

## Introduction

Despite wide acceptance in medical research, implementation of the FAIR (findability, accessibility, interoperability, and reusability) data principles in certain health domains and interoperability across data sources remain a challenge [1]. Clinical trial registries, such as ClinicalTrails.gov [2], the German Clinical Trials Register (DRKS) [3], and the World Health Organization’s International Clinical Trials Registry Platform (ICTRP) [4], collect metadata about clinical studies, thus making them searchable and findable [5]. However, they are often not sufficiently detailed and lack further information about relevant study documents (eg, protocols, questionnaires, data dictionaries, etc), which hinders the findability and accessibility of such significant health resources [5]. Moreover, numerous epidemiological and public health studies remain unregistered [1,5], likely due to unfamiliarity with metadata standards and a general lack of a metadata-sharing culture [6,7].

Making valuable data from these studies available to the research community could potentially improve our understanding of essential risk factors associated with various diseases [1]. Several data-sharing initiatives have aimed at promoting the implementation of the FAIR principles. In the field of biobanking, the Minimum Information About Biobank Data Sharing (MIABIS) initiative is dedicated to standardizing metadata describing biobanks, thereby improving their interoperability and enabling the sharing of their valuable data and samples [8]. In cardiology, the joint European Union-Canada project established a cross-border data sharing and multicohort cardiovascular research platform, linking molecular, imaging, functional, and clinical data [9]. In epidemiology, Maelstrom Research provides a comprehensive catalog with search tools to support the discovery of data collected by epidemiological research networks and studies [10,11]. To our knowledge, an overarching search for clinical trials alongside epidemiological and public health studies is not yet available in existing search portals [5].

The National Research Data Infrastructure for Personal Health Data (NFDI4Health) is a nationally funded multipartner German project that seeks to optimize data sharing among the clinical, epidemiological, and public health research communities while preserving protection, privacy, and ethical regulations. This infrastructure for data from various health-related disciplines intends to support health research and facilitate the development of new and personalized preventive interventions and therapies, thereby improving population health [12,13]. With this aim, NFDI4Health develops data management workflows, ensuring that research data are properly organized, documented, and stored throughout its lifecycle. These workflows involve defining metadata standards, data classification systems, and data governance policies that facilitate the findability and accessibility of research data via the central NFDI4Health search portal. Entitled the German Central Health Study Hub (GCHSH) [5,14], the platform allows for the standardized publication of descriptive metadata from clinical, epidemiological, and public health studies. It connects to local data repositories and harvests their relevant metadata via the Local Data Hub (LDH) software of NFDI4Health [15,16], making such studies and their data findable and accessible through a single-entry point.

In this paper, we present the development, structure, and implementation of the NFDI4Health metadata schema (MDS), the model underlying the GCHSH and LDH. We also present the ongoing mappings to other schemas, the format currently adopted, and the steps taken toward a machine-readable version. Prospects for further development and extension of the MDS are also discussed.

## Methods

### Ethical Considerations

As the study presented in this paper did not involve human participants and did not examine human personal data, the approval of an ethics committee was not required.

### NFDI4Health Structures and Their Roles in the Development of the MDS

**Figure 1:**
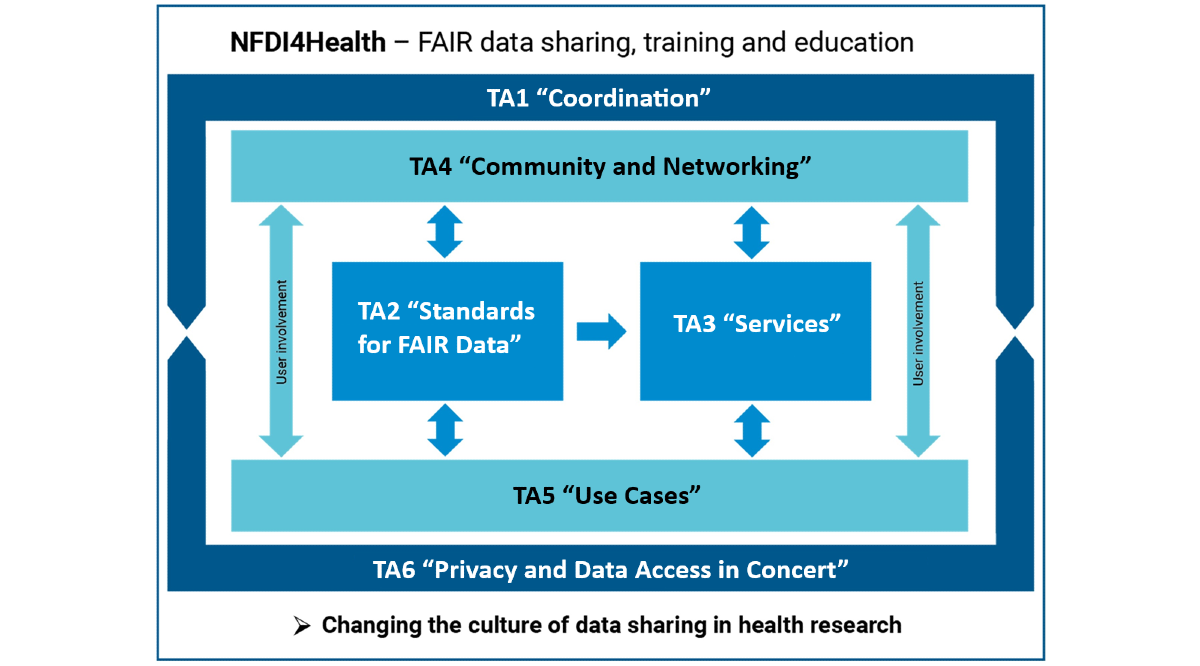
Tasks areas of the NFDI4Health. FAIR: findability, accessibility, interoperability, and reusability; NFDI4Health: National Research Data Infrastructure for Personal Health Data Project.

The NFDI4Health project comprises 6 task areas (TAs) [17] as shown in Figure 1, three of which are most relevant for the construction, development, and implementation of the MDS: TA2 “Standards for FAIR data,” TA3 “Services,” and TA5 “Use cases.” MDS development is a crucial part of TA2, as it aims to harmonize and further develop standards for data and their corresponding metadata in clinical trials, public health surveys, and epidemiological cohorts by implementing the FAIR principles. In that sense, TA2 provides the conceptual background for the NFDI4Health services implemented by TA3. Two crucial services that build on the metadata structures of the MDS are the GCHSH [14] and LDH [15,16], in addition to others that NFDI4Health aims to provide to the user community. All NFDI4Health standards and services are developed in close collaboration with domain experts and potential users in TA5 and implemented and tested within the scope of its 5 use cases, namely nutritional epidemiology, chronic diseases, record linkage, clinical trials, and medical imaging radiomics [18]. An additional use case focusing on COVID-19 was introduced during the pandemic, serving as a pilot and addressing the major challenges posed to societies around the world [19].

The MDS team (TA2) develops the metadata model as a conceptual backbone for NFDI4Health services and acts as the interface between NFDI4Health users (TA5) and developers (TA3). It is composed of experts in metadata as well as data interoperability and quality standards from different NFDI4Health coapplicant institutions. The team’s major role is to gather and bundle requirements from the users, model them into the schema, and communicate them to the developers for implementation. The team is also involved in various mapping activities between the MDS and other schemas, aiming to ensure the interoperability of the NFDI4Health metadata and services with other resources and platforms. Further interoperability efforts include representing the MDS using the Health Level 7 Fast Healthcare Interoperability Resources (FHIR) standard as well as value set mappings to concepts from international terminologies [20,21]. The MDS team holds Zoom meetings twice a month to coordinate, review, and discuss potential updates and improvements of the MDS and arrange for meetings with other teams (eg, developers, users, etc) when needed.

In addition to the 3 TAs directly involved in the construction and implementation of the MDS, TA6 “Privacy and data access in concert” provides the legal framework for metadata and data sharing. It helps in identifying relevant metadata items that capture information on data sharing, licensing, and access for studies and data addressed by NFDI4Health. This is especially important for future MDS extensions covering these aspects. TA4 “Community and networking” supports the outreach to the user community at large to demonstrate the usefulness of the MDS and its implementations to researchers and users in Germany and beyond. Finally, TA1 coordinates the whole NFDI4Health project and provides project management support.

### MDS Development

The MDS is an information model tailored to describe clinical, epidemiological, and public health studies as well as associated documents (eg, study protocols, instruments, etc) or datasets. It improves the FAIRness of these resources by making them searchable and accessible through implementations in metadata repositories tailored to the users’ needs, such as the GCHSH [14] and the LDH [15,16]. The MDS was initially developed by the NFDI4Health task force COVID-19 [19], including a single module modeled in Excel and aimed at COVID-19 studies [22]. Many items were primarily adapted from established metadata standards and models, including DataCite [23], ClinicalTrails.gov [2], DRKS [3], Maelstrom [10], MIABIS [24], and the Data Documentation Initiative (DDI) [25].

**Figure 2:**
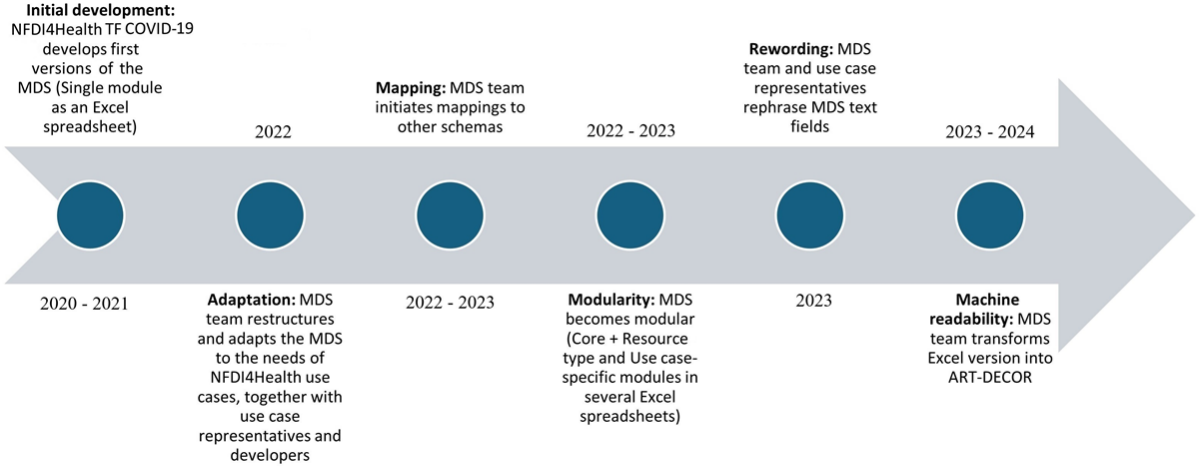
Development process of the MDS. ART-DECOR: Advanced Requirement Tooling-Data Elements, Codes, OIDs, and Rules; MDS: Metadata schema; NFDI4Health: National Research Data Infrastructure for Personal Health Data; TF: task force.

To ensure broad applicability and ease its implementation across further health domains, the MDS was restructured to meet the needs of other NFDI4Health use cases. It adopted a modular structure, comprising core and use case–specific modules. The extension was carried out by the MDS team in close collaboration with the service developers and use case representatives as domain experts. Mapping activities to other schemas were also initiated to facilitate interoperability. Upon using the GCHSH implementation of the MDS, feedback was received from the German Centers for Health Research, the coverCHILD project [26], among others, and further issues with the MDS were fixed. These included additional structural changes, improvements of item headers and description texts (rewording), and more standardized value sets. Transformation plans were finally executed toward an improved machine-readable version of the MDS in ART-DECOR (Advanced Requirement Tooling-Data Elements, Codes, OIDs, and Rules) [27]. The MDS development process from the year 2020 to 2024 is illustrated in Figure 2.

### Release Cycle

**Figure 3:**
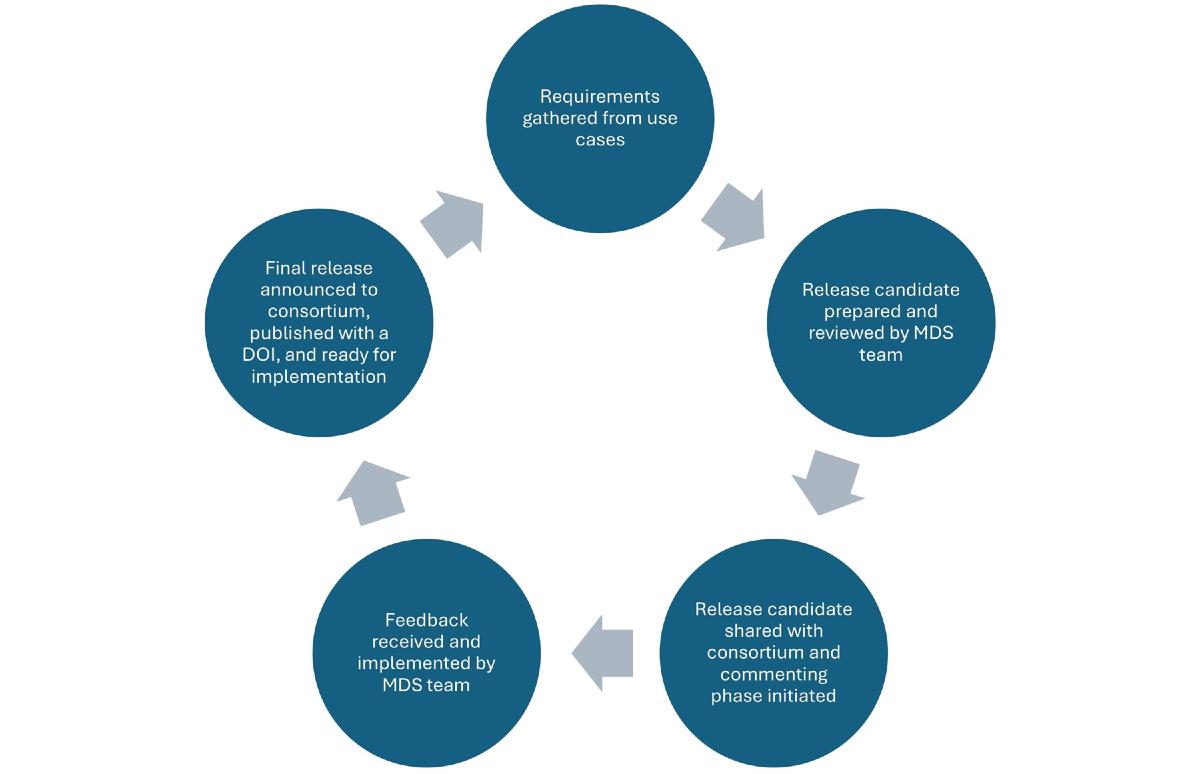
Release cycle of the MDS. DOI: digital object identifier; MDS: metadata schema.

To adapt to the requirements of the NFDI4Health user community, a new version of the MDS was released every 3 months. During each cycle, use case requirements were gathered and modeled and a release candidate of the MDS was prepared. Upon review by all members of the MDS team, the release candidate was shared with the NFDI4Health community and a commenting phase was initiated. During this phase, feedback could be provided by all members of the NDFI4Health consortium, including users and developers, commonly via a MDS GitHub repository. Feedback was also often provided via email, dedicated meetings, or during workshops. The feedback served as a basis to finalize the new version of the MDS, which was subsequently published with a digital object identifier (DOI) and shared with the GCHSH developers for implementation. The release cycle is shown in Figure 3.

### Mappings

For compatibility with existing clinical trial registries, the MDS was mapped to the study-specific models used by ClinicalTrails.gov [2], DRKS [3], and ICTRP [4]. Mappings were performed manually by comparing the MDS to the information present on these search portals, their input masks, as well as their XML exports and documentation, when available. The objective was to enable the GCHSH, and other services that implement the MDS, to automatically upload the metadata of studies from Germany (ie, studies with at least one participating facility in Germany) registered on these portals, thereby avoiding the need to re-enter their information. Accordingly, all mandatory items of the MDS were compared with individual items in these schemas in search of appropriate matches. Matches were identified by comparing names, definitions, and value sets. When matches were not found or not applicable, a fixed value was assigned to the mandatory field as a workaround for implementation.

To remain aligned with international and local initiatives, further mappings to the schemas of the European Clinical Research Infrastructure Network (ECRIN) [28,29] and the schema of the German Human Genome-Phenome Archive (GHGA) [30] were performed. These mappings were considered important because ECRIN supports the organization of clinical trials in Europe and GHGA develops one of the most relevant resources in Germany for genomic data from the health domain. The MDS team compared all mandatory items using the same approach described above. It was also agreed with both ECRIN and GHGA teams to prepare their own mapping version in the reverse direction, thereby comparing their mandatory items to the MDS in search of the best matches. The intention was to align both versions, with the goal of allowing the NFDI4Health services to exchange information with ECRIN’s metadata repository [31] and GHGA’s metadata catalog [32]. In addition, to examine alignment options with the European Rare Disease Registry Infrastructure (ERDRI), a discussion with colleagues from the European Commission’s Joint Research Center was initiated, followed by a comparison of the MDS items to the different sections of ERDRI’s directory of registries (ERDRI.dor) [33].

### Representations

The MDS was initially released as human-readable Microsoft Excel spreadsheets [34]. To develop an improved machine-readable version, a training workshop took place in May 2023 to kick-start a transformation into ART-DECOR [27]. Following the workshop, the Excel version of the MDS was shared with the ART-DECOR team to transform the modules’ main information, using their transformation script, into a dedicated ART-DECOR project. The transformed version was afterwards checked by various members of the MDS team. Transformation errors were identified and fixed, and missing information was manually added. In the context of the MDS representation in FHIR, allowed values were mapped to concepts from international terminologies, whenever possible, and integrated into ART-DECOR. MDS values that could not be matched to terminology concepts were placed in a local code system created for NFDI4Health. Besides the ART-DECOR representation, a Simplifier project was also established based on the MDS-to-FHIR mappings, in which different FHIR artifacts were created and published [35]. These, together with details on the terminology mappings, are outside the scope of this study and will be published elsewhere [36].

### Rewording

To improve user experience (UX), the GCHSH was tested by external users specialized in UX testing. Recommendations affecting the MDS included rephrasing item display names as well as descriptions and short input help texts based on the guidelines for effective UX writing [37]. Accordingly, dedicated discussions were initiated, involving NFDI4Health users from different domains as well as service developers and members of the MDS team, to examine the GCHSH interface item by item and individually modify and improve the clarity of these texts. Suggested changes were edited directly in a real-time working version of the MDS, which was shared on the NFDI4Health document management system for collaborative review and feedback.

## Results

### Structure of the Metadata Schema

**Figure 4:**
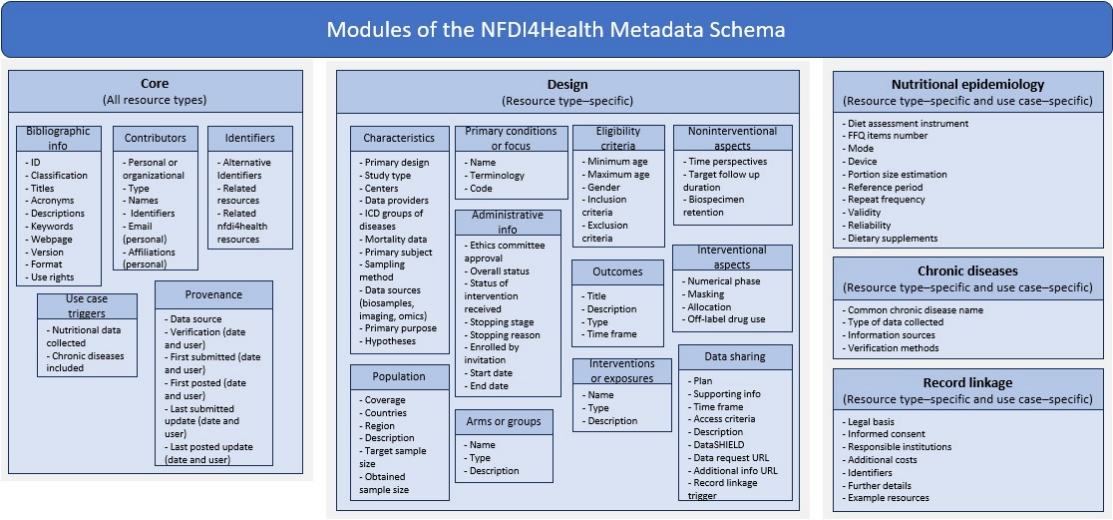
Overview of the NFDI4Health metadata schema modules. NFDI4Health: National Research Data Infrastructure for Personal Health Data.

The most recently released version of the MDS (v3.3) comprises a total of 220 metadata items in 5 dedicated modules. The core module captures information relevant to any type of health resource, while additional modules gather descriptions specific to particular resources, health domains, or use cases. The latter currently include nutritional epidemiology, chronic diseases, and record linkage. In NFDI4Health, the term “resource” or “resource type” is used to refer to health studies, substudies, registries, biobanks, study protocols, data dictionaries, among other types, data collection instruments, or study documents. All modules comprise mandatory and optional items, with conditional cardinalities set when needed. The core and design modules are mostly based on international standards, with 20 of their items representing the mandatory information required for entering or displaying studies on the GCHSH. The 3 use case–specific modules are user-driven, mainly based on the needs of the NFDI4Health user community. Figure 4 provides an overview of the different MDS modules, illustrating their corresponding item groups and subgroups. The complete list of items is available on the web [34].

#### Core Module

Based on DataCite, ClinicalTrails.gov, and DRKS, the core module of the MDS comprises 80 generic metadata items descriptive of any type of health resource. These include bibliographic information, such as the resource’s title, description, acronyms, keywords, and web page, along with information about the persons and organizations that contributed to the development of the resource. With the use of identifiers, the module provides links to published study results, in addition to relevant resources registered on the NFDI4Health portal or elsewhere. Provenance details about the publication of the resource as well as items to trigger the nutritional epidemiology and chronic diseases modules, where applicable, are also included.

#### Design Module

For design and data access information, the MDS provides 111 items, representing further characteristics that are specific to certain resource types, namely studies, substudies, and registries. Like the core module, the design module is also based on DataCite, ClinicalTrails.gov, and DRKS, in addition to Maelstrom, MIABIS, and DDI. For studies and substudies, the module distinguishes between interventional and noninterventional study designs and provides dedicated sections for each design type. Information about the study conditions is provided, in addition to a population section including recruitment area and sample size information. An administrative information section includes metadata items about the ethics committee approval, status, and dates of the study, while further sections cover details about the eligibility criteria, outcome measures, and time points. Information about the data sharing plan is also included, with an item to trigger the use case–specific record linkage module, when applicable. Most sections in this module also apply to registries due to several overlapping characteristics.

#### Nutritional Epidemiology Module

Dedicated to studies and substudies collecting nutritional data, this module provides 15 domain-specific items, mainly related to dietary assessment instruments used in corresponding studies. The modes and devices used to apply an instrument are specified in this module, along with the methods used for portion size estimation. The module also contains items indicating whether the instrument is repeatedly applied, whether it is validated and whether it inquires about dietary supplements.

#### Chronic Diseases Module

For each chronic disease addressed by the resource, this use case–specific module specifies whether prevalent or incident outcome data were collected and indicates the information sources from which the data were generated. Methods used to verify the outcomes are also described. The module contains 5 items and is only applicable to certain resource types, including study protocols, informed consent forms, case report forms, and patient information sheets, among other study documents.

#### Record Linkage Module

For resources allowing record linkage, the third dedicated module indicates whether specific legal regulations or permissions are required for conducting record linkage and whether informed consent has been obtained. The module also provides information about responsible institutions that must approve record linkage and whether additional costs are incurred. Identifiers that can be used (eg, date of birth, postal code, insurance number, etc) as well as DOIs of additional related resources specific to record linkage (eg, publications) are also included. The module has 9 items and is only applicable to studies, substudies, and registries.

### Preliminary Mapping Results

Based on the mappings of the MDS to the schemas of clinical trial registries, the GCHSH is capable of automatically uploading and displaying study metadata from these portals. With this objective, the 20 mandatory items of the MDS core and design modules were mapped to their counterparts in these schemas as shown in Table 1, while presetting the NFDI4Health resource type to “Study” and the country to “Germany,” as the focus of NFDI4Health is on German studies. The data source and subject items were also preset to “Automatically uploaded” and “Person,” respectively. This is considering that these studies would be imported into the GCHSH from clinical trial registries involving only human subjects. Items with value sets (eg, study type) were fixed to selected values such as “Unknown” or “Other,” as proper matching values could not be found. Moreover, fixed values, such as “Summary is not provided,” were used for string items (eg, description), as corresponding items or their texts were missing. Table 1 also includes mapping metrics, indicating the number of MDS items achieving various matching levels. By October 2024, there were 25595, 659, and 221 studies imported into the GCHSH from ClinicalTrails.gov, DRKS, and ICTRP respectively.

Following a similar approach and with the same objective, several iterations were prepared by the MDS team to map the MDS to version 6 (v6) of the ECRIN schemas [28]. As ECRIN has a study and a data object schema, those iterations were performed twice while considering the same study-specific items in Table 1 in addition to an extra item required for nonstudy resources (Resource.classification.typeGeneral). Table 2 shows mappings of the MDS mandatory items to both ECRIN schemas. Unlike the MDS, which includes only one scientific unabbreviated title for a resource, ECRIN considers several title types (eg, display title, abbreviated title, public title, subtitle, etc). This is also the case for the description item of a data object, which includes multiple types in ECRIN (eg, summary, abstract, table of contents, etc). Further, ECRIN distinguishes between contributors and creators while the MDS includes only contributors; yet, with contributor types matching with both categories in ECRIN. The MDS classifies contributor types into personal and organizational, while ECRIN includes a single list of contribution types, with matches to both groups in the MDS. The resource’s subject seems to be missing a direct match in ECRIN, yet could possibly be extracted from multiple items or topic types. Similarly, the data sharing plan does not seem to have a single corresponding item in ECRIN indicating whether data will or will not be shared, or if it is yet to be decided. However, items in ECRIN, such as the data sharing statement, access type, and access details could possibly include this information.

**Table 1:** Mappings of the mandatory items of the metadata schema core and design modules to the schemas of clinical trial registries.

NFDI4Health metadata schema (mandatory items) or Matching level		ClinicalTrails.gov	DRKS^a^	ICTRP^b^
**Core module**				
	Resource.classification.type	Fixed to “Study”	Fixed to “Study”	Fixed to “Study”
	Resource.titles.text	Official title	Title	Scientific title
	Resource.titles.language	Fixed to “EN (English)”	Fixed to “EN (English)”	Fixed to “EN (English)”
	Resource.descriptions.text	Detailed description	Brief summary in scientific language	Fixed to “Summary is not provided”
	Resource.descriptions.language	Fixed to “EN (English)”	Fixed to “EN (English)”	Fixed to “EN (English)”
	Resource.contributors.nameType	Responsible party type	Inferred from below fields	Inferred from below fields
	Resource.contributors.organizational.type and Resource.contributors.organizational.name	Responsible party, study sponsor, and collaborators	Primary sponsor, sources of monetary or material support	Primary sponsor, secondary sponsors
	Resource.contributors.personal.type, Resource.contributors.personal.givenName, and Resource.contributors.personal.familyName	Trial contacts, responsible party, and investigators	Contact for scientific queries, contact for public queries, and principal investigator	Contacts
	Resource.provenance.dataSource	Fixed to “Automatically uploaded: ClinicalTrials.gov”	Fixed to “Automatically uploaded: DRKS”	Fixed to “Automatically uploaded: ICTRP”
**Design module**				
	Design.primaryDesign	Study type	Study type	Study type
	Design.studyType.interventional	Interventional model	Assignment	Study design (fixed to “Unknown”)
	Design.studyType.nonInterventional	Observational model	Study type noninterventional, longitudinal or cross-sectional (fixed to “Other”)	Study design (fixed to “Unknown”)
	Design.groupsOfDiseases.generally	Fixed to “Unknown”	Fixed to “Unknown”	Fixed to “Unknown”
	Design.administrativeInformation.status	Recruitment status (combined with study dates)	Recruitment status	Recruitment status (fixed to “Other”)
	Design.subject	Fixed to “Person”	Fixed to “Person”	Fixed to “Person”
	Design.population.countries	Location countries (fixed to “Germany”)	Fixed to “Germany”	Countries of recruitment (fixed to “Germany”)
	Design.dataSharingPlan.generally	IPDSharing	IPD-individual participant data	Fixed to “Undecided, it is not yet known if data will be made available”
**Mapping metrics, n (%)**				
	Direct matches^c^	7 (35)	6 (30)	2 (10)
	Partial matches^d^	6 (30)	6 (30)	6 (30)
	Preset items (mapping not required)	4 (20)	4 (20)	4 (20)
	Fixed to a fitting value and could not be mapped	3 (15)	4 (20)	8 (40)

^a^German Clinical Trials Register.

^b^ICTRP: International Clinical Trials Registry Platform.

^c^Matched information included in one item in both the MDS and the corresponding matched schema.

^d^Matched information included in more than one item in the MDS, the corresponding matched schema, or both.

**Table 2:** Mappings of the mandatory items of the metadata schema core and design modules to the schemas of other initiatives.

NFDI4Health metadata schema (mandatory items) or Matching level			ECRIN^a^ Study Schema (v6)		ECRIN Data Object Schema (v6)		GHGA^b^		ERDRI^c^
**Core module**									
	Resource.classification.type	Fixed to “Study”		Type		Fixed to “Study”		Fixed to “Registry”	
	Resource.classification.typeGeneral	N/A^d^		Class		N/A		N/A	
	Resource.titles.text	Study titles (title text), when title type=Trial registry title		Object titles (title text)		Study.title		Name	
	Resource.titles.language	Study titles (language code)		Object titles (language code)		—^e^		—	
	Resource.descriptions.text	Brief description		Description (description text)		Study.description		Description	
	Resource.descriptions.language	—		Description (language code)		—		Fixed to “EN (English)”	
	Resource.contributors.nameType	—		Creators (name type), contributors (name type)		—		—	
	Resource.contributors.organizational.type	—		Contributors (contribution type)		—		Institution, sponsor	
	Resource.contributors.organizational.name	—		Creators (organization_organization default name)		Study.affiliations			
	Resource.contributors.personal.type	—		Contributors (contribution type)		—		Contact person	
	Resource.contributors.personal.givenName	—		Creators (person details_given name)		—			
	Resource.contributors.personal.familyName	—		Creators (person details_family name)		—			
	Resource.provenance.dataSource	Fixed to “Automatically uploaded: Other”		Fixed to “Automatically uploaded: Other”		Fixed to “Automatically uploaded: Other”		Fixed to “Automatically uploaded: Other”	
**Design module**									
	Design.primaryDesign	Study type		N/A		—		N/A	
	Design.studyType.interventional	Study features (intervention model)		N/A		Study.type (fixed to “Other”)		N/A	
	Design.studyType.nonInterventional	Study features (observational model)		N/A		Study.type (fixed to “Other”)		N/A	
	Design.groupsOfDiseases.generally	—		—		Condition title, condition name		*ICD-10* (*International Statistical Classification of Diseases, Tenth Revision*) code, disease name	
	Design.administrativeInformation.status	Study status		N/A		—		N/A	
	Design.subject	Study topics, when topic type=organism or subject characteristics (fixed to “Person”)		Topics, when topic type=subject characteristics (fixed to “Person”)		Fixed to “Person”		Fixed to “Person”	
	Design.population.countries	Fixed to “Germany”		Fixed to “Germany”		Fixed to “Germany”		Country (fixed to “Germany”)	
	Design.dataSharingPlan.generally	Data sharing statement		Access type, access details		—		Availability for future collaborations or studies	
**Mapping metrics, n (%)**									
	Direct matches^f^	4 (19)		2 (10)		3 (14)		3 (14)	
	Partial matches^g^	4 (19)		11 (52)		1 (5)		6 (29)	
	Preset and N/A items (mapping not required)	5 (24)		7 (33)		5 (24)		9 (43)	
	Fixed to a fitting value or could not be mapped	8 (38)		1 (5)		12 (57)		3 (14)	

^a^European Clinical Research Infrastructure Network.

^b^German Human Genome-Phenome Archive.

^c^ERDRI: European Rare Disease Registry Infrastructure.

^d^N/A: not applicable for the selected resource type or schema.

^e^Blank cells indicate that a match could not be found in this iteration.

^f^matched information included in one item in both the MDS and the corresponding matched schema.

^g^matched information included in more than one item in the MDS, the corresponding matched schema, or both.

Although Table 2 presents only the mapping results for the MDS mandatory items, the iterations also included comparisons of optional items and identified a few items in ECRIN that had no corresponding counterparts in the MDS. These include an item defining interstudy relationships, a composite item indicating the type of record keys used within a dataset, an item indicating the amount of deidentification applied to a dataset, and the European Open Science Cloud categorization recommended for data objects. Further mapping iterations have been performed to update the above mappings to ECRIN’s most recent version 8. The latest version has been shared with ECRIN colleagues as they were requested to prepare their own mapping version in the reverse direction. Detailed results of these mappings, along with value set comparisons, will be completed and published elsewhere.

Though still in the early stages, single iterations were also conducted by the primary author, mapping the mandatory items of the MDS to GHGA’s schema [30] and to the different attributes on the ERDRI.dor portal [33]. At the first glance, some commonalities among study-related items were identified and 9 of the 20 mandatory items of the MDS were preset or matched successfully to items in GHGA, as shown in Table 2. Remaining items could not be matched, as the distinction between interventional and noninterventional study designs in the MDS had no counterpart in GHGA’s schema, as well as the specification of the language used for study titles and descriptions. The controlled vocabularies used in GHGA’s schema were also not indicated in the version shared with the MDS team, making it challenging to match value set items. This first mapping iteration was shared with GHGA colleagues for review, in preparation of a second iteration. The MDS comparison to the different sections of ERDRI.dor revealed some matches among the registry-specific items as shown in Table 2. These, however, are yet to be shared with Joint Research Center colleagues for further discussion.

### Representation and Transformation Into ART-DECOR

The Excel version of the MDS [32] comprises individual spreadsheets for each module. Within each spreadsheet, items are listed in different rows and named according to the camelCase naming convention. Item names, data types, cardinalities, and allowed values are represented in multiple columns. Additional columns contain the information displayed for each item on the GCHSH. These include display names or headers, descriptions, additional descriptive information, input help texts, and input examples. Further spreadsheets include the sources used to build the MDS, value definitions, and documentation of all changes compared with the previous version. The authors of the MDS are also listed in a cover sheet, together with the persons responsible for the version, license details, and version information.

**Figure 5:**
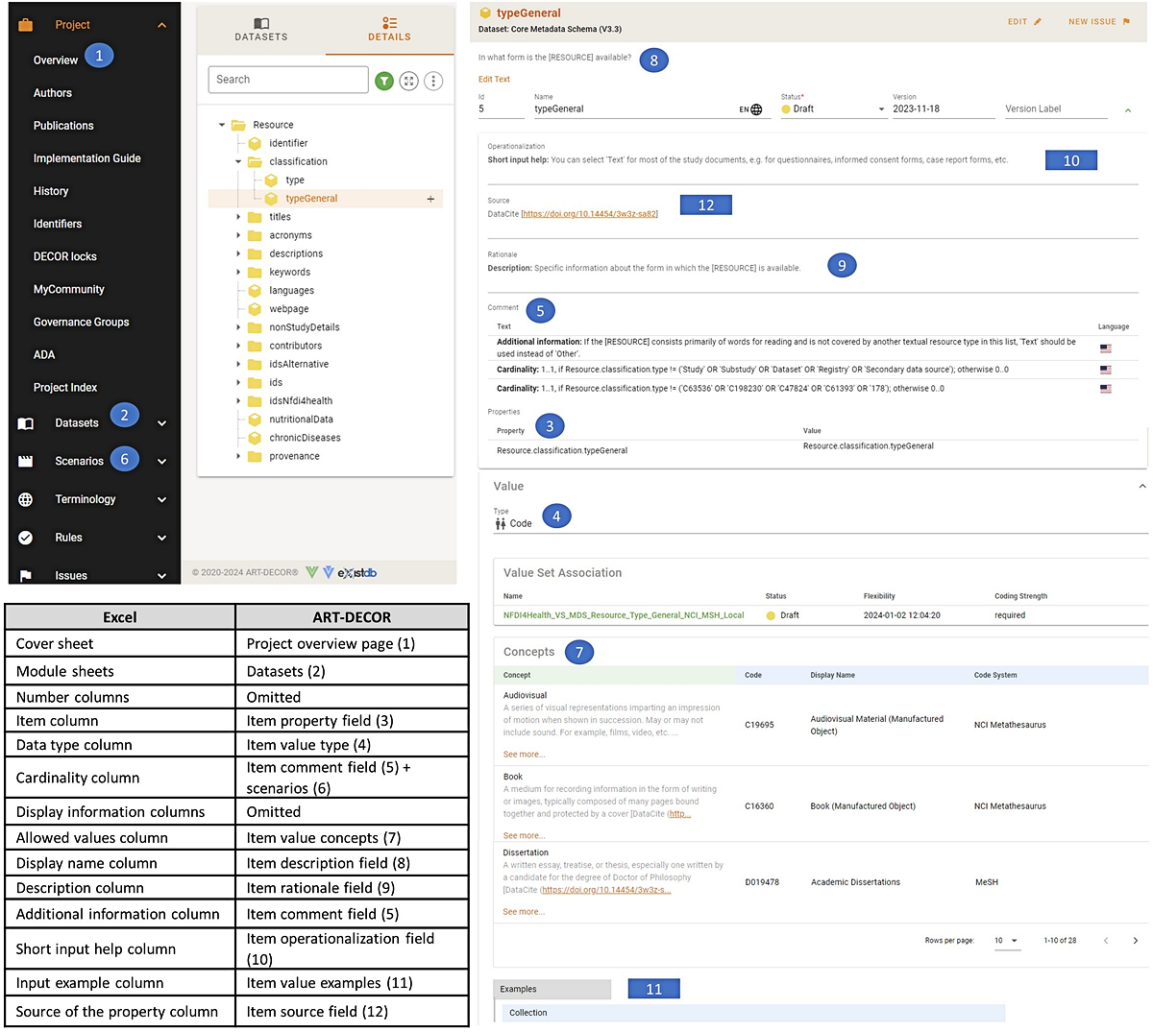
Excel to ART-DECOR transformation with a dataset item view. ART-DECOR: Advanced Requirement Tooling-Data Elements, Codes, OIDs, and Rules.

The transformation into ART-DECOR organizes the different modules from the Excel version into separate ART-DECOR datasets, with individual items arranged in a hierarchical tree structure. For each item in a particular dataset, the different Excel columns are represented in corresponding fields, including the item’s name, data type, and allowed values or concepts. The value definitions were directly added to the concept description field, thereby providing a structured list of concepts and their definitions. Cardinalities and conditions were initially added as free text in the comment field. However, they were later additionally implemented in different ART-DECOR scenarios toward a more structured version. Besides cardinalities, conformances are also specified in scenarios, indicating mandatory, optional, and conditional items. Figure 5 provides a dataset item view in ART-DECOR, together with a tabular list of Excel spreadsheets and columns and their corresponding representation in ART-DECOR. Terminology mappings are also illustrated under the section “Concepts,” listing the MDS item values and their matching terminology concepts. These mappings were taken from the value sets created previously to represent the MDS in FHIR.

To navigate the ART-DECOR project, the black menu on the upper left side of Figure 5 provides a list of different pages. The landing or overview page [27] of the project includes a brief description of the MDS, its authors, and copyright and license information. The datasets page enables adding new modules and displays the module hierarchies with all item information. The scenarios page allows for adding and viewing cardinalities and conditions for each dataset, yet in our project, these are also included in comments in the dataset view as shown in Figure 5. The publications page lists published versions of the MDS which have been finalized and can no longer be changed. The project index page allows for exporting the different datasets into various machine-readable formats (eg, XML). Finally, issues to be considered for the next versions of the MDS are raised and discussed on the issues page. Accordingly, the content viewed on these pages changes occasionally as the MDS is updated. Remaining pages on the menu are for technical use and are outside the scope of this paper. Further information on using ART-DECOR can be found in the tool’s documentation guide [38].

### Rephrasing of Display Texts

With 220 items to be examined, we could only check a few items from the core module by going through the search interface of the GCHSH. This exercise played a great role though in helping users associate the different columns of the Excel spreadsheets with what they usually see on the user interface. Further suggestions were provided via the real-time working version, yet many items were still not covered. This was probably due to the unfamiliarity of the users with the spreadsheets, making it overwhelming and time-consuming to go through the different columns for each item. The MDS team then worked independently on the real-time version and prepared several rewording iterations, covering 302 text changes in line with the UX guidelines [37]. The changes were clearly marked and users were asked to review them, provide their feedback, and agree to final changes in a dedicated meeting. The new texts were implemented in the latest version of the MDS [34].

### Implementation

The MDS is implemented in the GCHSH [14,39]. Upon creating an account, users are able to use the platform’s input mask or its application programming interfaces to register their resources. Information is requested based on the different sections of the MDS, including general information about the resource, its design characteristics, contributors, administrative information, and related resources. Mandatory and optional items are also indicated. The usage of the service has continuously increased, reaching in October 2024 a total of 626 resources manually registered on the portal, mostly by NFDI4Health users and the coverCHILD project. The majority of these resources were studies (n=243), data dictionaries (n=143), questionnaires (n=78), substudies (n=49), and other data collection instruments (n=35). Remaining resources included datasets (n=19), manuals of operations (SOPs; n=15), other study documents (n=15), registries or secondary data sources (n=9), case report forms (n=5), codebooks (n=5), interview schemes and themes (n=4), data management plans (n=3), other resource types (n=2), and statistical analysis plans (n=1). Based on their experience entering their metadata, users regularly report their issues via GitHub, email communication, or using the platform’s feedback button. Further development of the platform is performed interactively based on the received feedback. The platform is freely accessible to external users, thereby enriching its content and facilitating the collection of additional feedback to further improve user experience.

To facilitate sharing metadata by data holding organizations (DHOs), that is, the universities, research institutes, and data integration centers involved in conducting the studies, the MDS is also implemented by LDHs at 12 DHOs throughout Germany. Being the main local connector of the federated concept to the central services of NFDI4Health, the LDH ensures that local data are structured, linked, and easily shared among DHOs, while enabling them to automatically submit their metadata to the GCHSH [15,16]. The LDH also ensures adherence to the FAIR principles, allowing the discoverability of stored information, controlling data access, facilitating data exchange, and making data available for further use. The LDH software is currently rolled out in additional locations throughout Germany and internationally. Further applications of the MDS are promoted by the NFDI4Health FAIRsharing collection [40] of standards for health research data, incorporating standards used in the MDS development and promoting international interoperability.

## Discussion

### Principal Findings

This paper presented a MDS designed to capture and harmonize information from clinical, epidemiological, and public health studies, enhancing the findability and accessibility of their valuable data. The MDS is based on attributes from ClinicalTrails.gov [2], DRKS [3], Maelstrom [10], MIABIS [24], and DDI [25], in addition to the research needs derived from the NFDI4Health user community. Moreover, properties for assigning DOIs have been adopted from DataCite [23], thereby allowing the MDS to capture metadata about study documents (eg, questionnaires, data dictionaries, and electronic case report forms) and to enable persistent access to published resources. The MDS has the distinct ability to represent the hierarchical relation between different resources, including studies with complex designs and their associated documents. For instance, it can keep track of multiple studies in which a survey instrument is used or indicate that one study is part of another. The MDS also captures metadata essential for users to identify studies within specific health domains, such as studies collecting dietary intake or chronic disease information. It remains generic in its capacity to handle a broad range of resources such as datasets, registries, and data dictionaries, among others [5,41].

### Limitations and Challenges

#### Mapping Efforts and Updates

Due to the generic nature of the targeted schemas, mappings only focused on the core and design modules of the MDS. To illustrate the challenges of importing information into the GCHSH, only mappings of the MDS mandatory items were presented, with the use of fixed values when needed. Mappings were performed manually by comparing the various items of the mapped schemas in an Excel spreadsheet. Despite various efforts by several members of the MDS team, it remains challenging to keep clinical trial registry mappings up to date with newer versions of the MDS. This is mainly due to the absence of structured schemas for these portals, making it difficult for the MDS team members to agree on a common mapping approach or tool to identify matching items. As a result, the mappings presented in this paper are based on older versions of the MDS and need to be revised. Detailed updated mappings, also containing optional items and value sets of the MDS, will be published elsewhere.

As ECRIN’s study and data object schemas are based on ClinicalTrails.gov and DataCite respectively, identifying commonalities with the MDS was expected. However, despite their well-structured and detailed schemas, mapping the MDS to their items was still challenging due to their strict distinction between studies and data objects. The abundance of composite attributes and value sets in their schemas also often led us to recognize that some items in ECRIN could be represented by multiple items in the MDS and vice versa. For instance, the study titles item in ECRIN comprises text, type, language, and comment attributes, with the title type having multiple categories. The title item in the MDS only includes text and language and is defined as the scientific unabbreviated title of the study. This could be matched with multiple title type categories in ECRIN, including trial registry title, public title, protocol title, and other scientific title. Similarly, the study features item in ECRIN has categorized feature type and feature value attributes. Each feature type, together with its corresponding feature values, could be matched with a different group of items in the MDS (eg, masking, allocation, study type, primary purpose, etc). Matching items conditionally on certain titles or feature types presented a challenge to both the MDS and development teams. This became even more challenging when matching value sets, due to the presence of user-defined values in the MDS which lacked standardized sources or definitions. It was, therefore, suggested that a better approach could be to agree on a minimal common data model, thus allowing both platforms to exchange data while saving mapping efforts. The feasibility of this approach, however, is yet to be assessed.

Though still in the early stages, our initial mapping attempt to the GHGA schema also had its challenges. This was mainly due to the strict classification of studies in the MDS by design (ie, interventional vs noninterventional) as well as its support of multiple languages for study titles and descriptions. It was not clear whether such a classification existed in the GHGA schema and if more than one language could be used to provide study descriptions. Further, due to technical issues accessing their public version, we could not identify which controlled vocabularies were used in their schema for codable items, thus limiting value set mappings.

Similarly, mapping to the ERDRI.dor schema is still at an early stage. As ERDRI.dor is a platform for registering European rare disease registries, it is not surprising that some commonalities with our MDS registry-specific items could be detected. Yet, due to the lack of a structured schema, these commonalities could only be observed by examining the different sections of the ERDRI.dor platform. For accurate mappings, further discussions, particularly with ERDRI’s metadata team, remain needed.

#### Excel Versus ART-DECOR Representation

With over 200 items incorporated in the latest MDS releases, it became cumbersome for the MDS team to maintain and frequently update the Excel version and document all changes in a trackable manner. It also became difficult for the users to follow the frequent updates and provide their feedback. Following several discussions about alternative representation formats, the ART-DECOR open-source tool was selected. Due to its user-friendly interface, the tool enables efficient use by experts with or without a technical background, thus allowing NFDI4Health users, developers, and members of the MDS team to work collaboratively on the MDS developments. The tool’s major advantage over Excel is that it represents the modeled data elements in a hierarchical structure, thereby illustrating the relations between the various items in an accessible manner. Unlike Excel, the tool also offers diagram views of all modules as well as exports to various machine-readable formats such as XML, JavaScript Object Notation, and FHIR R4. This in turn facilitates the transformations needed for the GCHSH development and representing the model in Simplifier and keeping the FHIR profiling up to date.

Despite these numerous advantages and the continued support from the ART-DECOR team, the transformation from Excel to ART-DECOR faced several obstacles. Though scenarios allowed us to set cardinalities, conformances, and textual and structured conditions, setting cross-module structured cardinality conditions (ie, conditions involving items from different modules) was not permitted. This left us only with the option of using textual conditions in scenarios for better machine readability while keeping the comment field in the datasets for better clarity and human readability.

Another issue we experienced during the transformation was the representation of the mappings to other schemas. Currently, the tool does not offer a structured way for this purpose. This once again left us only with the possibility of adding mappings as free text in the comment field. As this would still require multiple efforts when newer versions of the mapped schemas are released, we skipped representing the mappings in this version and decided to start a discussion with the ART-DECOR team about introducing a new mapping feature to the tool. Many user-defined values in the MDS could not be mapped to terminology concepts and we could not trace them back to determine why they were included and whether there was a need to keep them. This is because the specific sources for each value were not documented in earlier versions of the MDS during development. Placing them in a local code system was agreed upon as a temporary solution until finding proper matches, requesting their introduction to appropriate terminologies, or deciding to remove them from the MDS altogether.

### Conclusions and Future Work

This paper presented the development, structure, and representations of the NFDI4Health MDS. The MDS aims to improve the representation of data from clinical, epidemiological, and public health studies, thereby enhancing their FAIRness. With its generic nature and modular structure, it is also transferable to adjacent research fields. The MDS was developed in close collaboration with the NDFI4Health use case experts, thereby ensuring that the needs of the greater user community are met. In addition to its core and design modules, the MDS currently provides 3 use case–specific modules. These cover the domains of nutritional epidemiology, chronic diseases, and record linkage. Two additional modules for clinical trials and imaging radiomics data will soon be implemented. The latest version of the MDS (v3.3) was released in Excel format and transformed into ART-DECOR. Yet, toward better machine readability, future versions will only be released in ART-DECOR.

The MDS is implemented in the GCHSH and several NFDI4Health LDHs. This allows users to register their metadata either centrally in the GCHSH or locally in a LDH instance of their own institution for automatic integration into the GCHSH. For better interoperability with other study portals, the MDS has been mapped to the schemas of clinical trial registries, thereby allowing the automatic upload of their metadata to the GCHSH. To ensure alignment with international and local initiatives, mappings to the ECRIN schemas, ERDRI, and the GHGA schema are ongoing. Mapping of the MDS to the health-related extension of the DCAT-AP metadata standard (HealthDCAT-AP) is also planned, thereby preparing the NFDI4Health services for interoperability with the emerging European Health Data Space.

Having focused on the core and design modules so far, mappings need to extend to the use case–specific modules and strategies need to be defined for handling user-defined items and values in the MDS, as well as items with no counterparts in either of the mapped schemas. To facilitate these activities, mapping goals and standardized mapping approaches need to be agreed upon in NFDI4Health, thus defining common procedures for matching items, limiting mapping efforts, and optimizing the results of mapping activities performed by different members of the MDS team. In addition, identifying tools that support manual mapping activities could help extend and automize mappings to other data models. To facilitate alignment with international initiatives, the MDS also needs to increase its focus on terminology-based value sets and adopt to a larger extent existing terminologies and FHIR resources, when applicable. For these reasons, the MDS remains in development and is constantly expanded based on the needs of the user community.

## Abbreviations

ART-DECOR: Advanced Requirement Tooling-Data Elements, Codes, OIDs, and Rules
DDI: Data Documentation Initiative
DHO: data holding organizations
DOI: digital object identifier
DRKS: German Clinical Trials Register
ECRIN: European Clinical Research Infrastructure Network
ERDRI: European Rare Disease Registry Infrastructure
ERDRI.dor: European Rare Disease Registry Infrastructure, directory of registries
FAIR: findability, accessibility, interoperability, reusability
FHIR: Fast Healthcare Interoperability Resources
GCHSH: German Central Health Study Hub
GHGA: German Human Genome-Phenome Archive
ICTRP: International Clinical Trials Registry Platform
LDH: Local Data Hub
MDS: metadata schema
MIABIS: Minimum Information About Biobank Data Sharing
NFDI4Health: National Research Data Infrastructure for Personal Health Data
TA: task area
UX: user experience
