# Correction: Toward a Domain-Overarching Metadata Schema for Making Health Research Studies FAIR (Findable, Accessible, Interoperable, and Reusable): Development of the NFDI4Health Metadata Schema

**DOI:** 10.2196/78151

**Published:** 2025-06-04

**Authors:** Haitham Abaza, Aliaksandra Shutsko, Sophie A I Klopfenstein, Carina N Vorisek, Carsten Oliver Schmidt, Claudia Brünings-Kuppe, Vera Clemens, Johannes Darms, Sabine Hanß, Timm Intemann, Franziska Jannasch, Elisa Kasbohm, Birte Lindstädt, Matthias Löbe, Katharina Nimptsch, Ute Nöthlings, Marisabel Gonzalez Ocanto, Tracy Bonsu Osei, Ines Perrar, Manuela Peters, Tobias Pischon, Ulrich Sax, Matthias B Schulze, Florian Schwarz, Carolina Schwedhelm, Sylvia Thun, Dagmar Waltemath, Atinkut A Zeleke, Wolfgang Müller, Martin Golebiewski

**Affiliations:** 1 Scientific Databases and Visualization Heidelberg Institute for Theoretical Studies (HITS) Heidelberg Germany; 2 Information Centre for Life Sciences German National Library of Medicine (ZB MED) Cologne Germany; 3 Core Facility Digital Medicine and Interoperability Universitätsmedizin Berlin Berlin Institute of Health at Charité Berlin Germany; 4 Institute for Community Medicine University Medicine Greifswald Greifswald Germany; 5 Department of Biometry and Data Management Leibniz Institute for Prevention Research and Epidemiology - BIPS Bremen Germany; 6 Department of Medical Informatics University Medical Center Göttingen Georg-August-University Göttingen Germany; 7 Department of Molecular Epidemiology German Institute of Human Nutrition Potsdam-Rehbruecke Nuthetal Germany; 8 Institute for Medical Informatics, Statistics and Epidemiology University Leipzig Leipzig Germany; 9 Molecular Epidemiology Research Group Max-Delbrück-Center for Molecular Medicine in the Helmholtz Association (MDC) Berlin Germany; 10 Department of Nutrition and Food Science University of Bonn Bonn Germany

In “Toward a Domain-Overarching Metadata Schema for Making Health Research Studies FAIR (Findable, Accessible, Interoperable, and Reusable): Development of the NFDI4Health Metadata Schema” (JMIR Med Inform 2025;13:e63906) the following corrections have been made.

Author Hannes Wünsche was erroneously included in the authorship list. Hence, their name and affiliation have been removed.

In addition, the following affiliation has been changed from:

Department of Molecular Epidemiology, German Institute of Human Nutrition, Potsdam-Rehbruecke, Nuthetal, Germany

to:

Department of Molecular Epidemiology, German Institute of Human Nutrition Potsdam-Rehbruecke, Nuthetal, Germany

The corrections will appear in the online version of the paper on the JMIR Publications website, together with the publication of this correction notice. Because this was made after submission to PubMed, PubMed Central, and other full-text repositories, the corrected article has also been resubmitted to those repositories.

